# Immune responses to the adjuvanted recombinant zoster vaccine in immunocompromised adults: a comprehensive overview

**DOI:** 10.1080/21645515.2021.1930846

**Published:** 2021-06-30

**Authors:** Alemnew F. Dagnew, Peter Vink, Mamadou Drame, David O. Willer, Bruno Salaun, Anne E. Schuind

**Affiliations:** a GSK, Rockville, MD, USA; b GSK, Mississauga, Ontario, Canada; c GSK, Rixensart, Belgium

**Keywords:** Humoral immunity, Cell-mediated immunity, Immune response, Adjuvanted recombinant zoster vaccine, Immunocompromised

## Abstract

Immunocompromised (IC) persons are at increased risk for herpes zoster (HZ) and its complications, mainly due to impairment of cell-mediated immunity (CMI). The adjuvanted recombinant zoster vaccine (RZV) demonstrated efficacy against HZ in autologous hematopoietic stem cell transplant (auto-HSCT) recipients and hematologic malignancy (HM) patients. We review immune responses to RZV in 5 adult IC populations, 4 of which were receiving multiple, concomitant immunosuppressive medications: auto-HSCT and renal transplant recipients, HM and solid tumor patients, and human immunodeficiency virus-infected adults. Although administered in most cases when immunosuppression was near its maximum, including concomitantly with chemotherapy cycles, RZV induced robust and persistent humoral and, more importantly, CMI responses in all 5 IC populations. Based on the overall clinical data generated in older adults and IC individuals, RZV is expected to provide benefit in a broad adult population at risk for HZ.

## Introduction

Herpes zoster (HZ) results from reactivation of latent varicella-zoster virus (VZV) and may occur at any age. Although the incidence of HZ and its complications increases with age, it is higher in immunocompromised (IC) persons regardless of age.^1–4^ In addition, both HZ and its complications tend to be more severe and last longer in IC individuals.

IC populations are heterogeneous and can have different levels of immune suppression depending on age, underlying disease, and the type, duration, and combination of immunosuppressive therapies.^5^ The increased risk of HZ in IC populations may be critically influenced by the impairment of cell-mediated immunity (CMI) resulting from these specific medical conditions, their treatments, or both.^1,2^

The risk of HZ in IC populations can be mitigated by vaccination. Whereas live-attenuated vaccines are contraindicated in IC populations because of the risk of disseminated disease,^6,7^ non-live HZ vaccines have been evaluated in IC adults with the highest incidences of HZ, including chronically immunosuppressed renal transplant (RT) recipients,^8^ hematopoietic stem cell transplant (HSCT) recipients,^9–12^ hematologic malignancy (HM) patients,^12,13^ and solid tumor (ST) patients.^12,14^ Non-live HZ vaccines have also been evaluated in human immunodeficiency virus (HIV)-infected adults, another IC population at increased risk of HZ.^12,15^

HIV infection induces immunosuppression that directly affects CD4 T-cells. Accordingly, while the incidence of HZ ranges between 3–5/1000 person-years in the general population,^16^ the incidence in HIV-infected individuals reached 32/1000 person-years in the pre-antiretroviral therapy (ART) era, and remains at 10–11/1000 person-years, even in the ART era.^4,17,18^

Solid organ transplant recipients represent a distinct group of IC patients. To prevent allograft rejection, their immune systems are suppressed by chronic therapies, resulting in a mixed immunodeficiency, consisting mainly of T-cell-mediated immunity impairment.^19^ HZ incidences up to 22–32/1000 person-years have been reported in recipients of various solid organ transplants.^4,20–23^

HSCT recipients are IC as a result of pre-transplant conditioning regimens, which eradicate the disease and create space for engraftment, but also increase the risk of HZ. The risk is generally highest during the first year following transplantation, with incidences of 8%-25% in autologous HSCT (auto-HSCT) recipients^24–27^ and 13%-28% in allogenic HSCT recipients.^28,29^ HZ risk decreases within 2–3 years post-HSCT as the transplant engrafts, matures, and reconstitutes the immune system.^4,24,30^

The risk for HZ is also increased in cancer patients under treatment, of whom those with HMs appear to be most susceptible.^4^ In patients receiving immunosuppressive cancer therapies for HMs or STs, incidences up to 31/1000 and 14/1000 person-years, respectively, have been reported.^31,32^

The increased HZ risk shows that there is a clear medical need to prevent HZ in IC populations.

Although an investigational inactivated VZV vaccine demonstrated efficacy against HZ in auto-HSCT recipients and ST patients, it was not efficacious in HM patients^11,33^ and has not been licensed to date.

The adjuvanted recombinant zoster vaccine (RZV, *Shingrix*, GSK) is a recombinant VZV glycoprotein E (gE) non-live subunit vaccine that cannot cause disseminated HZ. RZV is highly immunogenic and ≥90% efficacious in preventing HZ in all age groups among adults ≥50 years.^34,35^ Efficacy is also maintained in individuals with underlying medical conditions,^36^ including potential immune-mediated diseases.^37^ RZV has received licensure for use in adults ≥50 years of age (YOA) in many countries since 2017, and in adults ≥18 YOA at increased HZ risk in Europe since 2020.

RZV was well tolerated in auto-HSCT and RT recipients, HM and ST patients, and HIV-infected adults.^38^ Efficacy of RZV was demonstrated in auto-HSCT recipients^10^ and HM patients.^13^ Here we present a comprehensive overview of immune responses to RZV in these 5 severely IC populations.^8,10,13–15,39^

## Methods

### Design of the reviewed studies with RZV in IC populations

All 5 studies were randomized, observer-blind, placebo-controlled, and parallel-group studies (Table 1). The study protocols were reviewed and approved by relevant institutional review boards or independent ethics committees.

**Table 1.** Overview of the reviewed clinical studies with RZV in immunocompromised adults

Population and schedule	Study countries and years and clinicaltrials.gov registration numbers	Summary of study design and objectives	Participants included in the ATP cohort for humoral immunogenicity
HIV-infected adults ≥18 YOA stratified in 3 subgroups: ART/high CD4 (ART duration ≥1 year, viral load <40 copies/mL, CD4 T-cell count ≥200 cells/mm^3^), ART/low CD4 (ART duration ≥1 year, viral load <40 copies/mL, CD4 T-cell count 50–199 cells/mm^3^), ART-naïve/high CD4 (ART-naïve and ART not anticipated to be used until after 1 month post-last dose, viral load 1000–100,000 copies/mL, and CD4 T-cell count ≥500 cells/mm^3^) 3 doses (at months 0, 2, and 6)	Germany, United Kingdom, United States 2010–2013 NCT01165203	Phase I/IIa, randomized, observer-blind, placebo-controlled study.^15^ Objectives included evaluation of safety (including hematology and biochemistry parameters and worsening of HIV condition), humoral and cell-mediated immunogenicity, and occurrence, duration, and severity of HZ cases and complications.	3 RZV doses: 54 3 placebo doses: 37
Autologous HSCT recipients ≥18 YOA (ZOE-HSCT) 2 doses (at months 0 and 1–2) with the first dose administered within 50–70 days after transplant	Australia, Belgium, Bulgaria, Canada, Czech Republic, Estonia, Finland, France, Germany, Greece, Hong Kong, Israel, Italy, Japan, Malaysia, the Netherlands, New Zealand, Panama, Poland, Romania, Russian Federation, South Africa, South Korea, Spain, Taiwan, Turkey, United Kingdom, United States 2012–2017 NCT01610414	Phase III, randomized, observer-blind, placebo-controlled efficacy study.^10,39^ Minimization according to the underlying disease (multiple myeloma or other diagnoses [including non-Hodgkin B-cell lymphoma, Hodgkin lymphoma, non-Hodgkin T-cell lymphoma, acute myeloid leukemia, solid organ malignancies, etc.]). Objectives included evaluation of vaccine efficacy in preventing HZ and its complications, safety, and humoral and cell-mediated immunogenicity.	2 RZV doses: 82 2 placebo doses: 76
Hematologic malignancy patients ≥18 YOA 2 doses (at months 0 and 1–2) with administration at ≥10 days before or after a chemotherapy cycle or 10 days to 6 months after completion of the full chemotherapy course	Australia, Belgium, Canada, Czech Republic, Finland, France, Hong Kong, Italy, New Zealand, Pakistan, Panama, Poland, Russian Federation, Singapore, South Korea, Spain, Sweden, Taiwan, Turkey, United Kingdom, United States 2013–2017 NCT01767467	Phase III, randomized, observer-blind, placebo-controlled study.^13^ Stratification according to underlying disease (non-Hodgkin B-cell lymphoma, chronic lymphocytic leukemia, or other diseases [multiple myeloma, non-Hodgkin T-cell lymphoma, Hodgkin lymphoma, and other hematologic malignancies]). Objectives included evaluation of safety, humoral and cell-mediated immunogenicity, and incidence of confirmed HZ cases. Efficacy was evaluated post hoc.	2 RZV doses: 217 2 placebo doses:198
Solid tumor patients ≥18 YOA on chemotherapy 2 doses (at months 0 and 1–2) with first dose at 8–30 days before the first (occasionally second) cycle of a chemotherapy course (PreChemo) or at the start (±1 day) of and concurrently with a chemotherapy cycle (OnChemo); the second dose was administered to all participants at the first day (allowing a window of ±1 day) of a subsequent chemotherapy cycle	Canada, Czech Republic, France, South Korea, Spain, United Kingdom 2013–2016 NCT01798056	Phase II/III, randomized, observer-blind, placebo-controlled study.^14^ Stratification (4:1) in PreChemo or OnChemo stratum. Objectives included evaluation of safety and humoral and cell-mediated immunogenicity.	2 RZV doses: 87 2 placebo doses: 98
Renal transplant patients ≥18 YOA 2 doses (at months 0 and 1–2) with the first dose administered at 4–18 months post-transplant	Belgium, Canada, Czech Republic, Finland, Italy, Panama, South Korea, Spain, Taiwan 2014–2017 NCT02058589	Phase III, randomized, observer-blind, placebo-controlled study.^8^ Objectives included evaluation of safety (including allograft function and rejection), humoral and cell-mediated immunogenicity, and incidence of suspected HZ cases.	2 RZV doses:121 2 placebo doses:119

ART, antiretroviral therapy; ATP, according-to-protocol; HZ, herpes zoster; RZV, adjuvanted recombinant zoster vaccine; ZOE-HSCT, Zoster efficacy study in autologous hematopoietic stem cell transplant recipients; YOA, years of age.

### Participants

All 5 studies included participants ≥18 YOA without an HZ/varicella episode or vaccination within the year before the first dose. Administration of licensed live and other non-replicating vaccines were excluded for specific time periods. Women of childbearing potential could participate if practicing contraception from 30 days prior to study vaccination through 2 months (M) (HM and ST patients, RT recipients, HIV-infected adults) or 12 M (auto-HSCT recipients) post-dose 2.

The use of investigational or non-registered drugs/vaccines from 30 days prior to study vaccination to study end was not allowed in auto-HSCT and RT recipients, HM patients, and HIV-infected adults. However, investigational use of a registered (auto-HSCT recipients and HM patients) or non-registered (auto-HSCT recipients) product to treat the underlying disease was allowed. Other, study-specific inclusion/exclusion criteria have been described previously.^8,10,13–15^

### Procedures

RZV was administered intramuscularly as 3 doses at M0, M2, and M6 in HIV-infected adults,^15^ and as 2 doses 1–2 M apart in the other IC populations.^8,10,13,14^ Each dose contained recombinant VZV gE antigen (50 μg) and the AS01_B_ adjuvant system (containing 50 μg of 3-O-desacyl-4′-monophosphoryl lipid A, 50 μg of *Quillaja saponaria* Molina fraction 21 [licensed by GSK from Antigenics LLC, a wholly owned subsidiary of Agenus Inc., a Delaware, USA corporation] and liposome).

### Immunogenicity objectives and outcomes

Anti-gE antibody concentrations and frequencies of CD4[2+] T-cells (gE-specific CD4 T-cells expressing ≥2 activation markers from among interferon-γ, interleukin-2, tumor necrosis factor-α, and cluster of differentiation 40 ligand), and the vaccine response (VR) in terms of anti-gE antibody and CD4[2+] T-cells were evaluated in all populations. Results are presented for 1 M and 12 M post-last dose overall and, where available, per age group (18–49 and ≥50 YOA). Analyses per diagnoses (auto-HSCT recipients, HM patients), timing of vaccination in relation to the immunosuppressive therapy (HM and ST patients), or immunosuppressive regimen (RT recipients) were also performed. The second dose was the last in all IC populations except for HIV-infected adults, in whom the third dose was the last. Of note, no substantial benefit of the third over the second dose was seen in HIV-infected adults in terms of humoral or CMI responses.^15^ Finally, except for the study in auto-HSCT recipients, in which efficacy was the primary outcome, all studies had immunogenicity objectives with predefined statistical success criteria (Table 2).

**Table 2.** Immunogenicity objectives with predefined statistical success criteria

	2-dose RVZ schedule						3-dose RZV schedule	
	Hematologic malignancy patients		Solid tumor patients		Renal transplant recipients		HIV-infected adults	
Statistical success criterion	Objective type	Group/cohort	Objective type	Group/cohort	Objective type	Group/cohort	Objective type	Group/cohort
**Humoral immunity**								
The LL of the 95%CI (90% CI for HIV patients) of the (adjusted*) geometric mean ratio (RZV over Placebo) for anti-gE antibody concentrations >3 at 1 month post-last dose	Primary	All participants excluding NHBCL&CLL	Primary	PreChemo	Secondary	All participants	Primary	High CD4 (ART and ART-naïve)
	Secondary	All participants excluding NHBCL	Secondary	All participants				
The LL of the 95%CI of the VRR for anti-gE antibody concentrations in the RZV group ≥60% at 1 month post-last dose	Primary	RZV group excluding NHBCL&CLL	Secondary	RZV group of PreChemo	Primary	RZV group	-	-
	Secondary	RZV group excluding NHBCL	Secondary	RZV group of all participants				
**Cell-mediated immunity (CMI)**								
The LL of the 95%CI (70% CI for HIV patients) of the (adjusted**) geometric mean ratio (RZV over Placebo) for gE-specific CD4[2+] T-cell frequencies >1 (>2 for HIV patients) at 1 month post-last dose	-	-	Secondary	PreChemo CMI sub-cohort	Secondary	CMI sub-cohort	Primary	High CD4 (ART and ART-naïve)
The LL of the CI of the 95% VRR for gE-specific CD4[2+] T-cell frequencies in the RZV group ≥50% at 1 month post-last dose	-	-	Secondary	RZV group of PreChemo CMI sub-cohort	Secondary	RZV group CMI sub-cohort	-	-

*in hematologic malignancy and solid tumor patients, and in renal transplant recipients

**in solid tumor patients and renal transplant recipients

ART, antiretroviral therapy; CI, confidence interval; CLL, chronic lymphocytic leukemia; CMI, cell-mediated immunity; gE, glycoprotein E; HIV, human immunodeficiency virus; LL, lower limit; NHBCL, non-Hodgkin B-cell lymphoma; RZV, adjuvanted recombinant zoster vaccine, VRR, vaccine response rate (i.e., proportion of participants meeting the criterion for vaccine response).

### Main statistical considerations for immunogenicity assessment

Immunogenicity was evaluated in the according-to-protocol (ATP) cohorts for immunogenicity/persistence, which included study participants who complied with the protocol and had available immunogenicity data. The ATP cohorts for humoral immunogenicity originated from a subset (auto-HSCT recipients) or the entire study populations. The ATP cohorts for CMI originated from the entire study population (HIV-infected adults) or from subsets of study populations. The cohorts and statistical success criteria used for inferential analyses are presented in Table 2. For descriptive analyses, anti-gE antibody geometric mean concentrations (GMCs) were determined with their exact 2-sided 95% confidence intervals (CIs), and frequencies of CD4[2+] T-cells were tabulated using descriptive statistics (minimum, first quartile, median, third quartile, maximum).

Additional details about the assessment of anti-gE antibodies and CD4[2+] T-cells are presented elsewhere.^8,10,13–15,40^

## Results

### Study populations

Within each IC population, demographic characteristics were balanced between the RZV and Placebo groups in the ATP cohort for immunogenicity. Most RZV recipients were ≥50 YOA (70%, mean ages at first dose 46.5–57.0 YOA), male (58.5%-94.4%) except for ST patients (34.5% males), of White-Caucasian/European ancestry (66.1%-96.8%), and of non-Hispanic/Latino ethnicity (90.1%-100%).

In RZV recipients from the ATP cohorts for immunogenicity, 31.8% of HM patients were vaccinated during the cancer therapy course and 68.2% after the cancer therapy course, and 74.7% of ST patients were vaccinated PreChemo and 25.3% OnChemo (definitions in Table 1). The most frequent diagnoses were multiple myeloma (53.7%) in auto-HSCT recipients, breast (51.4%) and colorectal (21.1%) cancer in ST patients, and multiple myeloma (24.0%), Hodgkin lymphoma (18.0%), chronic lymphocytic leukemia (CLL, 16.6%), and non-Hodgkin B-cell lymphoma (NHBCL, 15.2%) in HM patients. Among RT recipients, 77.3% received a maintenance immunosuppressive regimen consisting of calcineurin inhibitors/sirolimus (CIS) + mycophenolate compound (MC) + corticosteroids (CS), 17.6% received CIS+MC, and 5.0% received CIS+CS.

When assessed in all vaccinated auto-HSCT recipients, the median time between transplant and first dose was 61.0 days.

### Humoral immune responses to RZV

Across the 5 IC populations, ≥95.1% of RZV recipients were seropositive for anti-gE antibody before vaccination,^41^ and pre-vaccination anti-gE antibody GMCs ranged between 763–1354 milli-international units per milliliter (mIU/mL).

**Figure 1.**
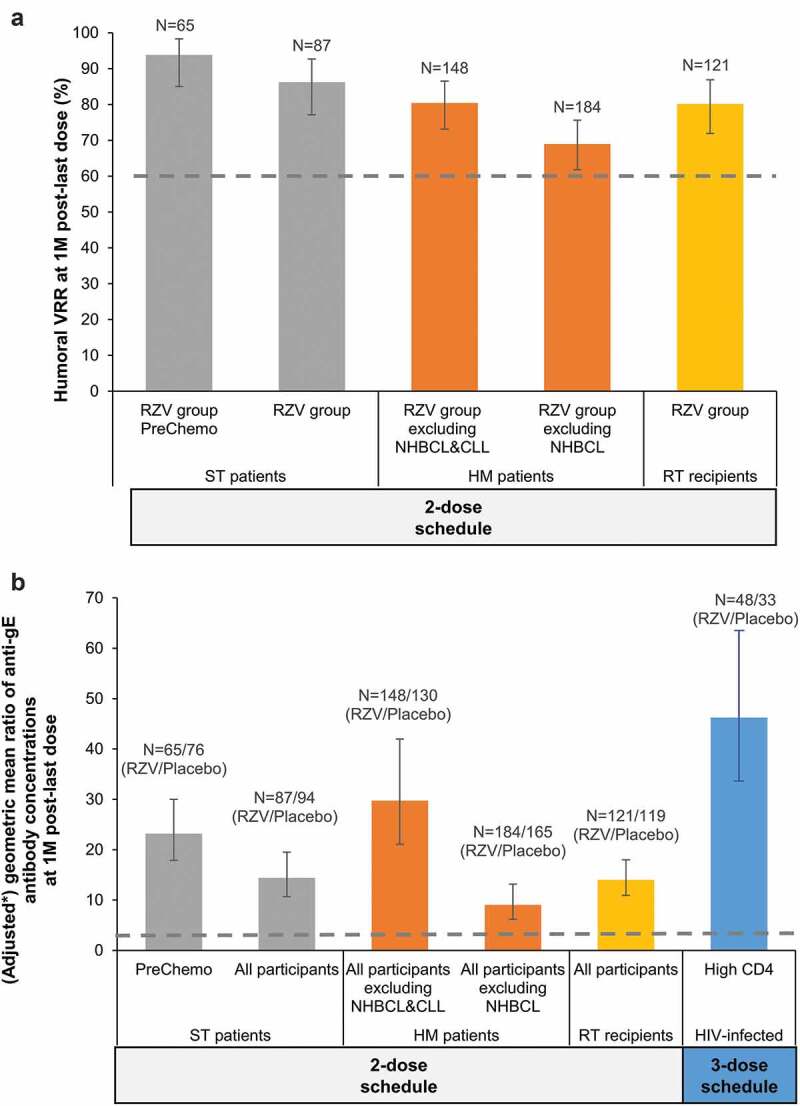
Inferential humoral immunogenicity analyses in the reviewed studies with RZV in immunocompromised populations.

Success criteria were met for all inferential humoral immunogenicity objectives (Figure 1).

CLL, chronic lymphocytic leukemia; gE, glycoprotein E; HIV, human immunodeficiency virus; HM, hematologic malignancy; M, month; N, number of participants; NHBCL, non-Hodgkin B-cell lymphoma; RT, renal transplant; RZV, adjuvanted recombinant zoster vaccine; ST, solid tumor; VRR, vaccine response rate, defined as the proportion of participants with a ≥ 4-fold increase in the anti-gE antibody concentration compared either with the pre-vaccination concentration (initially seropositive participants) or with the anti-gE antibody concentration cutoff value for seropositivity (initially seronegative participants); the cutoff for seropositivity was 97 milli-international units per milliliter (mIU/mL) in all populations except for HIV-infected adults, in whom it was 18 mIU/mL; **black error bars** depict two-sided exact 95% confidence intervals; the **blue error bar** (HIV-infected) depicts two-sided exact 90% confidence interval; **dashed lines** represent predefined statistical criteria (i.e. thresholds that need to be exceeded by the lower limits of the confidence intervals for objectives to be considered met). *in ST and HM patients, and in RT recipients

**Figure 2.**
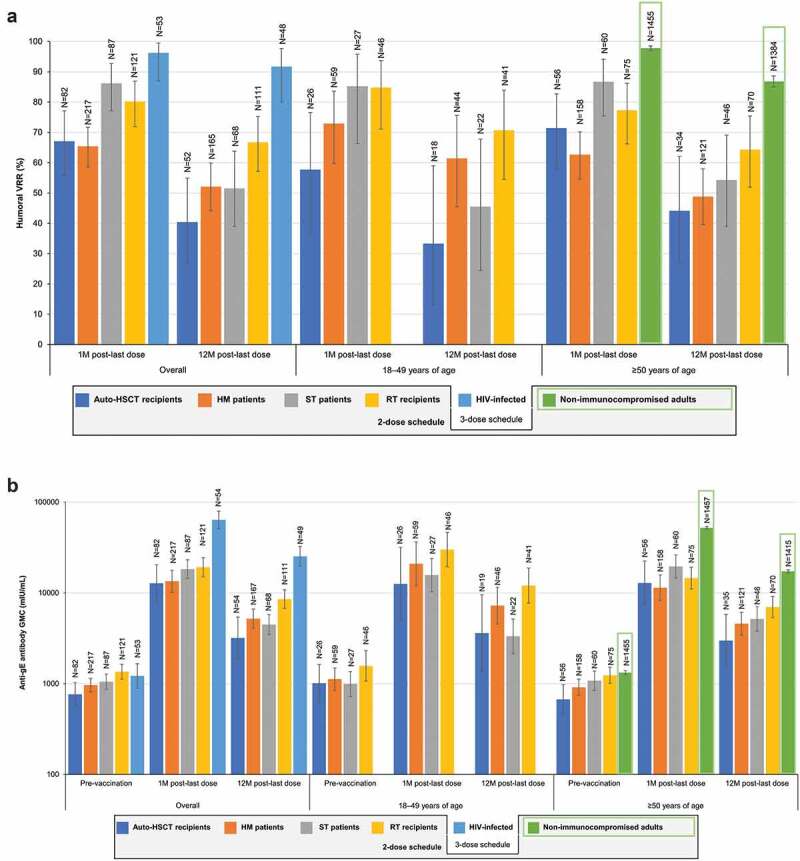
Humoral immune responses to RZV in immunocompromised populations.

At 1 M post-last dose, humoral VR rates (VRRs) ranged between 65.4%-96.2% across the 5 IC populations (Figure 2a). Anti-gE antibody GMCs at 1 M post-last dose ranged between 12,753–19,164 mIU/mL across IC populations, except for HIV-infected adults, in whom it was 63,813 mIU/mL; at 12 M post-last dose these were 3184–8545 and 25,242 mIU/mL (4.2–6.3 and 20.7 times higher than pre-vaccination), respectively (Figure 2b).

gE, glycoprotein E; GMC, geometric mean concentration; HIV, human immunodeficiency virus; HM, hematologic malignancy; HSCT, hematopoietic stem cell transplant; M, month; N, number of participants with available results; RT, renal transplant; RZV, adjuvanted recombinant zoster vaccine; ST, solid tumor; VRR, vaccine response rate, defined as the proportion of participants with a ≥ 4-fold increase in the anti-gE antibody concentration compared either with the prevaccination concentration (initially seropositive participants) or with the anti-gE antibody concentration cutoff value for seropositivity (initially seronegative participants). The cutoff for seropositivity was 97 milli-international units per milliliter (mIU/mL) in all populations except for HIV-infected adults, in whom it was 18 mIU/mL; error bars depict two-sided exact 95% confidence intervals. Note: the green rectangles represent findings in non-immunocompromised older adults,^40^ which are only included here for reference.

Age (18–49 or ≥50 YOA) did not appear to affect humoral immune responses in auto-HSCT recipients and ST patients (Figure 2). A slight trend for stronger humoral responses in the younger age group was seen in HM patients and RT recipients.

Auto-HSCT recipients and HM patients diagnosed with NHBCL had a diminished humoral immune response compared with other diagnoses, noting that 97% of HM patients with NHBCL were treated with rituximab. HM patients with CLL (of whom 81% were treated with rituximab) also had a diminished response. At 1 M post-last dose, humoral VRR was 14.2% in auto-HSCT recipients with NHBCL compared with 67.1% in the entire study population. Similarly, humoral VRR was 45.5% and 22.2% in HM patients with NHBCL and CLL, respectively, compared with 80.4% in the rest of the study population.

HM patients vaccinated after the cancer therapy course had a stronger humoral response than those vaccinated during the cancer therapy course. In ST patients, the humoral response to RZV was higher in the PreChemo than in the OnChemo subgroup (definitions in Table 1) at 1 M post-last dose but were similar at 12 M post-last dose. In RT recipients, humoral responses at 1 M and 12 M post-last dose 2 were similar regardless the type of chronic, daily immunosuppressive regimen (CIS+MC+CS, CIS+MC, or CIS+CS).

### CMI responses to RZV

In HIV-infected adults, the geometric mean ratio of CD4[2+] T-cell frequencies at 1 M post-dose 3 over 1 M post-dose 2 was 1.0 (95%CI: 0.8–1.3), showing that the third dose had no incremental benefit over the second. In the other 4 reviewed studies, performed after evaluating the 3-dose schedule in HIV-infected adults, 2 doses were administered.

**Figure 3.**
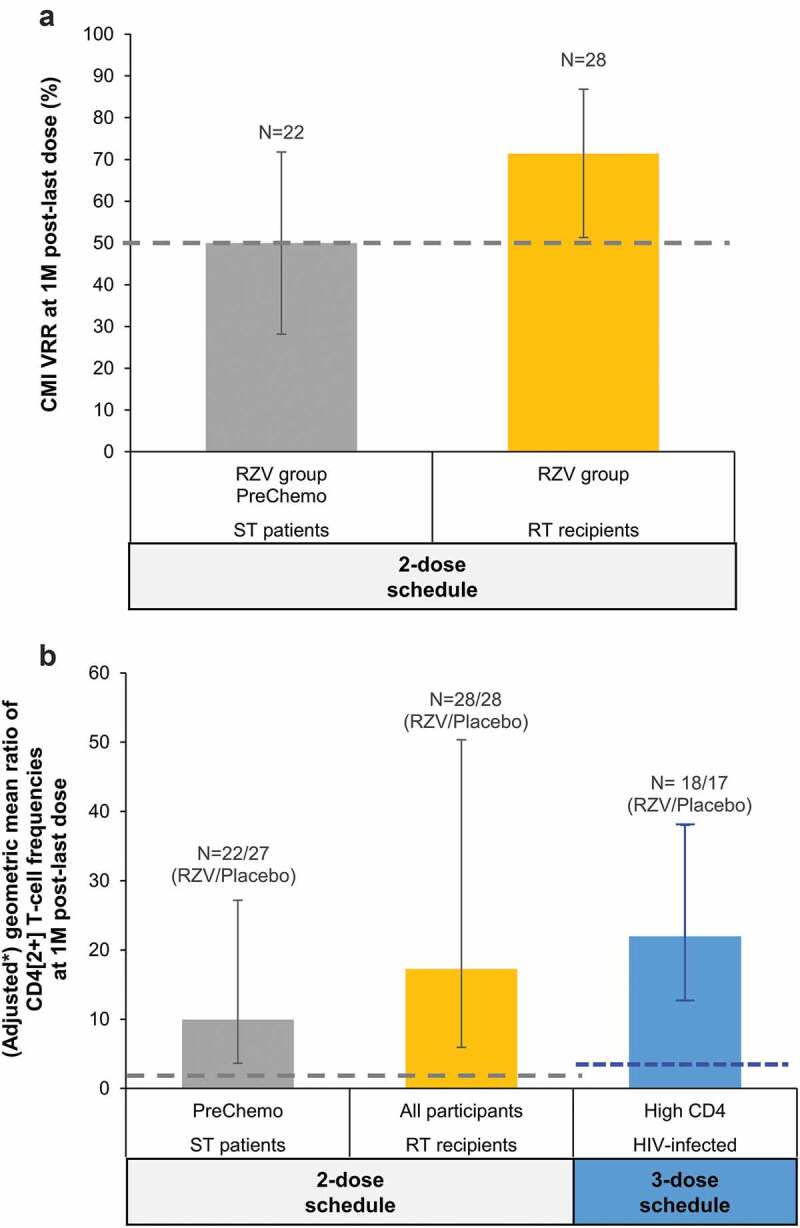
Inferential CMI analyses in the reviewed studies with RZV in immunocompromised populations.

All CMI objectives met predefined statistical success criteria in RT recipients and HIV-infected adults. In the PreChemo subgroup of ST patients, although higher CD4[2+] T-cell frequencies were demonstrated in RZV versus placebo recipients, the CMI VRR was 50.0% (95%CI: 28.2–71.8) in the RZV group, and the success criterion for this objective was not met (Figure 3).

CD4[2+] T-cells, gE-specific CD4 T-cells expressing ≥2 activation markers from among interferon-γ, interleukin-2, tumor necrosis factor-α, and cluster of differentiation 40 ligand; CMI, cell-mediated immunity; gE, glycoprotein E; HIV, human immunodeficiency virus; M, month; N, number of participants; RT, renal transplant; RZV, adjuvanted recombinant zoster vaccine; ST, solid tumor; VRR, vaccine response rate, defined as the proportion of participants with a ≥ 2-fold increase in the frequency of CD4[2+] T-cells compared with prevaccination frequency (for participants with prevaccination CD4[2+] T-cell frequency ≥320 per 10^6^ CD4 T-cells) or with the cutoff (for participants with prevaccination frequencies below the cutoff); **black error bars** depict two-sided exact 95% confidence intervals; the **blue error bar** (HIV-infected) depicts two-sided exact 70% confidence interval; **dashed lines** represent pre-defined statistical criteria (i.e., thresholds that need to be exceeded by the lower limits of the confidence intervals for objectives to be considered met). *in ST patients and RT recipients

**Figure 4.**
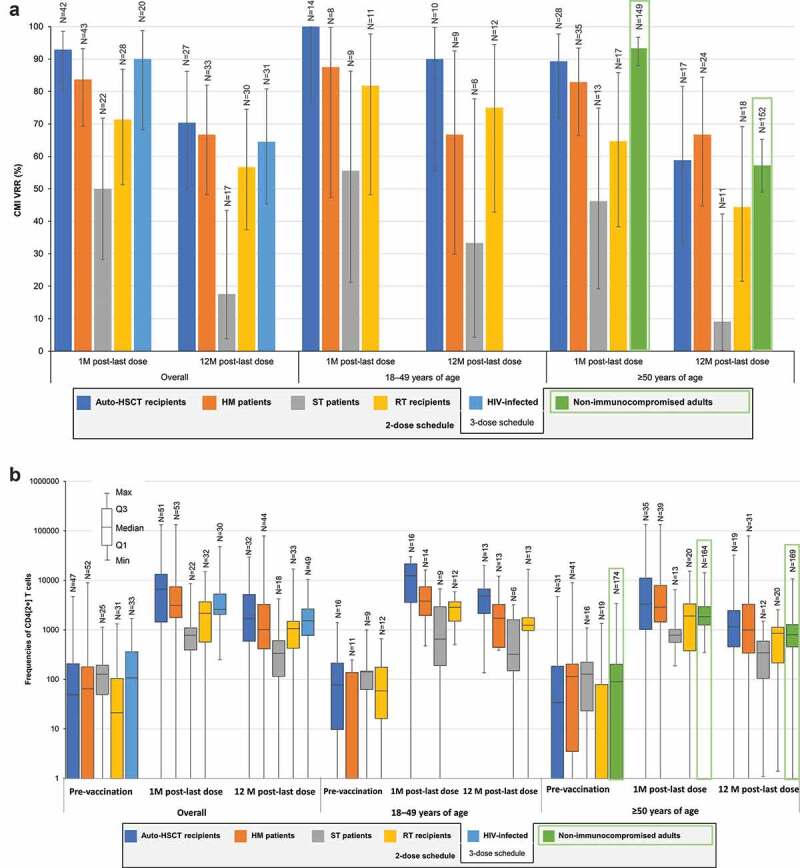
CMI responses to RZV in immunocompromised populations.

At 1 M post-last dose, CMI VRRs ranged between 71.4%-92.9% across IC populations, except for ST patients, as noted above (Figure 4a). Median pre-vaccination CD4[2+] T-cell frequencies ranged between 21 and 127 across the 5 IC populations. At 1 M post-last dose, median CD4[2+] T-cell frequencies ranged between 2149–6645 across IC populations, except for ST patients, in whom it was 779; at 12 M post-last dose, these were 1007–1706 and 333 (13.0–50.3 and 2.6 times higher than pre-vaccination), respectively (Figure 4b).

CD4[2+] T-cells, gE-specific CD4 T-cells expressing ≥2 activation markers from among interferon-γ, interleukin-2, tumor necrosis factor-α, and cluster of differentiation 40 ligand; CMI, cell-mediated immunity; gE, glycoprotein E; HIV, human immunodeficiency virus; HM, hematologic malignancy; HSCT, hematopoietic stem cell transplant; M, month; N, number of participants with available results; Q1, Q3, first and third quartiles; RT, renal transplant; RZV, adjuvanted recombinant zoster vaccine; ST, solid tumor; VRR, vaccine response rate, defined as the proportion of participants with a ≥ 2-fold increase in the frequency of CD4[2+] T-cells compared with prevaccination frequency (for participants with prevaccination CD4[2+] T-cell frequency ≥320 per 10^6^ CD4 T-cells) or with the cutoff (for participants with prevaccination frequencies below the cutoff); error bars depict two-sided exact 95% confidence intervals (panel A). Note: the green rectangles represent findings in non-immunocompromised older adults,^40^ which are only included here for reference.

Age (18–49 or ≥50 YOA) did generally not affect the magnitude of CMI responses in auto-HSCT, RT, ST, and HM populations (Figure 4).

Underlying diagnoses in auto-HSCT recipients and HM patients did not affect CMI responses. At 1 M post-last dose, the VRR was 100% in both auto-HSCT recipients and HM patients with NHBCL and 71.4% in HM patients with CLL, compared with 92.9% in all auto-HSCT recipients and 73.7% in HM patients excluding those with NHBCL or CLL. CMI responses tended to be higher in HM patients vaccinated after the cancer therapy course than in those vaccinated during cancer therapy.

## Discussion

**Figure 5.**
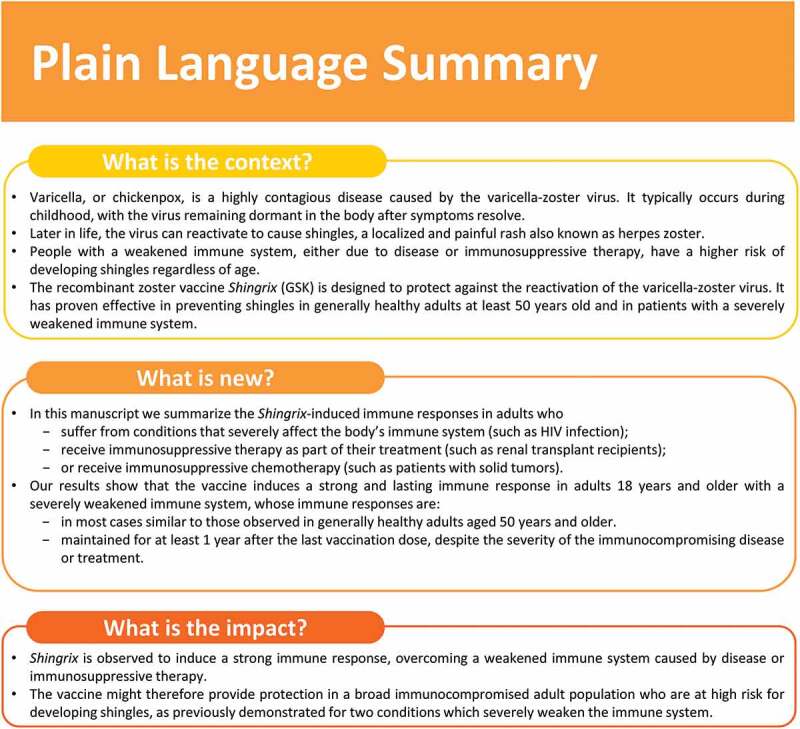
Plain language summary.

Prevention of viral infections, including the reactivation of latent VZV, in adults with immunodeficiency or immunosuppression caused by disease and/or its therapy is a large unmet medical need. Immunogenicity data show that RZV is able to induce robust and persistent gE-specific humoral and CMI responses in different populations, including adults with severely immunocompromising conditions and/or under immunosuppressive treatments. This demonstrates that, in addition to being able to overcome immunosenescence in adults ≥50 YOA,^40,42^ RZV can also overcome severe immunocompromising conditions. A plain language summary contextualizing the results and potential clinical research relevance and impact is presented in Figure 5.

Age did not affect efficacy of RZV in auto-HSCT recipients,^10^ and only had a limited effect on immune responses in IC populations with available age-based data. This has also been observed in non-IC adults, in whom efficacy and immune responses were similar across all age groups ≥50 years.^34,35,40^

High proportions (65%-96%) of IC study participants mounted a humoral VR to the RZV vaccination course, and anti-gE antibody concentrations increased substantially from pre-vaccination levels in all 5 IC populations ≥18 YOA, and were maintained above pre-vaccination levels through the end of a 1-year follow-up period.^8,10,13–15^

Some underlying diseases in HM patients and auto-HSCT recipients, specifically CLL and/or NHBCL, were associated with a less robust humoral response to RZV. This is likely due to B-cell depletion induced by anti-CD20 monoclonal antibody therapy (e.g., rituximab), which was frequently administered to HM patients with NHBCL and CLL.^13^ However, this severe B-cell impairment does not appear to affect the protection offered by RZV, because the efficacy of RZV against HZ was maintained at 60.5% (95%CI: 31.0–78.2) in auto-HSCT recipients with NHBCL.^39^

Unlike humoral immune responses, CMI responses to RZV were not affected by underlying diseases in IC populations, and were generally maintained through the end of a 1-year follow-up period at levels similar to those observed in non-IC adults ≥50 YOA (Figure 4). CMI responses are regarded as the main mechanistic driver for protection against HZ,^43^ and RZV demonstrated protection against HZ and postherpetic neuralgia (PHN) across different types of adult populations, including auto-HSCT recipients, which is the studied IC population at the greatest risk for HZ.^4,10,13,34–37^ Although the first RZV dose was administered early in immune reconstitution, 50–70 days post-auto-HSCT, RZV was 68.2% efficacious against HZ and 89.3% efficacious against PHN in the overall auto-HSCT population.^10^ As mentioned above, efficacy against HZ was observed in participants with any of the underlying diseases, including NHBCL (60.5%), despite the less robust humoral response.^39^ In a post hoc analysis, RZV also demonstrated 87.2% efficacy against HZ in HM patients, but the sample size did not allow assessment per individual underlying disease. Nevertheless, the results in auto-HSCT recipients suggest that RZV may remain efficacious in HM patients with NHBCL or CLL, in whom humoral responses were less robust.^13^ Although efficacy was not evaluated in ST patients and RT recipients, their CMI responses were robust and similar to those in auto-HSCT recipients and HM patients, suggestive of a similar clinical benefit.

Overall, the high CD4 T-cell responses observed in IC populations may result from the ability of the AS01 adjuvant system to promote a CMI response, regardless of age, underlying medical condition, or treatment.^44^ CD4 T-cell responses were strong in HIV-infected adults ≥18 YOA, and comparable to those observed in non-IC adults ≥50 YOA (Figure 4). This might be partly because most were receiving ART with immune reconstitution and had CD4 T-cell counts ≥200 cells/mm^3^.^15^ The CD4 T-cell response was particularly high in auto-HSCT recipients, which could be explained by the homeostatic proliferation following vaccination or by the low number of total CD4 T-cells, which is used as the denominator in the calculation of CD4[2+] T-cell frequencies. In auto-HSCT recipients, CD4 T-cell responses displayed polyfunctional profiles similar to those in adults ≥50 YOA,^39^ and polyfunctional responses after vaccines against tuberculosis, malaria, melanoma, or HIV have been shown to correlate with protection.^45–48^ While data for RZV-elicited CD8 T-cell responses are not available for IC populations,^8,10,13–15^ using the same CMI assessment methods, only scarce CD8 T-cell responses were detected in older adults, which did not increase upon vaccination with RZV.^40,42^

The use of cytotoxic immunosuppressive chemotherapy is expected to interfere with the generation of antigen-specific lymphocytes, particularly when vaccination is undertaken concurrently with a chemotherapy cycle. In ST patients, the first dose was administered either 8–30 days before the first (occasionally second) cycle of a chemotherapy course (PreChemo) or at the start of and concurrently with a chemotherapy cycle (OnChemo). The second dose was administered concurrently with a chemotherapy cycle in all the ST patients. Although in ST patients, CMI responses were less robust than in HM patients, to whom RZV was administered ≥10 days before or after a chemotherapy cycle or 10 days to 6 M after completion of the immunosuppressive chemotherapy course,^13^ humoral responses were in similar ranges. In ST patients, humoral immune responses tended to be higher in the PreChemo than in the OnChemo subgroup shortly after vaccination. The 8–30-day time window allowed PreChemo RZV recipients to develop an immune response to the first dose before immunosuppressive therapy was initiated, which likely accounts for this difference.^14^

In addition to the high efficacy observed in older adults,^34,35^ the efficacy of RZV was also remarkable in 2 populations that are highly IC and at the highest risk of HZ.^10,13^ Although vaccine efficacy cannot be inferred for all IC populations in the absence of a correlate of protection, the overall clinical data generated in older adults and IC individuals ≥18 YOA indicate that the anticipated benefit-risk profile of RZV for the prevention of HZ in adults is favorable.^39,49^

Limitations of studies evaluating immunogenicity of RZV in IC populations result from the heterogeneity of populations and of immunosuppressive treatments within studies. In some subgroups per age, underlying disease, immunosuppressive therapy, or timing of vaccination in relation to the immunosuppressive therapy, the number of participants was very small, especially for the evaluation of CMI. As for other vaccines, due to the ever-growing field of immunosuppressive therapies and standard of care, the most beneficial timing and dosing of RZV vaccination remains to be determined.

## Conclusion

RZV induced robust and persistent humoral and, more importantly, CMI responses in patients with a wide variety of IC conditions, most of whom were receiving multiple immunosuppressive medications. These include auto-HSCT recipients, who are at highest risk of HZ. In most cases, vaccination was undertaken when immunosuppression was near its maximum. Nonetheless, efficacy against HZ has been evaluated and demonstrated in auto-HSCT recipients and HM patients. Because the mechanisms leading to increased risk of HZ are believed to be shared between older adults (immunosenescent) and other adults at increased risk of HZ (individuals with IC conditions, treatments, autoimmune diseases, stress, depression, family HZ history), and because the treatments used in the IC populations studied are also used to treat other medical conditions, RZV is expected to benefit a broad adult population at risk for HZ by overcoming the CMI impairment, which is thought to be the basis for VZV reactivation.

## Data Availability

Anonymized individual participant data and study documents for the 5 reviewed studies can be requested for further research from www.clinicalstudydatarequest.com

## Trademark

Shingrix is a trademark of the GSK group of companies.
